# Deciphering New Players in the Neurogenic Adult Hippocampal Niche

**DOI:** 10.3389/fcell.2020.00548

**Published:** 2020-07-02

**Authors:** Antonela Bonafina, Gustavo Paratcha, Fernanda Ledda

**Affiliations:** ^1^División de Neurociencia Molecular y Celular, Instituto de Biología Celular y Neurociencias, Universidad de Buenos Aires, Consejo Nacional de Investigaciones Científicas y Técnicas, Buenos Aires, Argentina; ^2^Fundación Instituto Leloir, Instituto de Investigaciones Bioquímicas de Buenos Aires, Consejo Nacional de Investigaciones Científicas y Técnicas, Buenos Aires, Argentina

**Keywords:** neural stem cells, adult hippocampal neurogenesis, niche signals, adult born granule cells, granule cell integration

## Abstract

In the mammalian adult hippocampus, new neurons are continuously generated throughout life in the subgranular zone of the dentate gyrus. Increasing evidence point out the contribution of adult-born hippocampal granule cells (GCs) to cognitive processes such as learning and memory, indicating the relevance of understanding the molecular mechanisms that control the development of these new neurons in the preexisting hippocampal circuits. Cell proliferation and functional integration of adult-born GCs is a process highly regulated by different intrinsic and extrinsic factors. In this review, we discuss recent advances related with cellular components and extrinsic signals of the hippocampal neurogenic niche that support and modulate neurogenesis under physiological conditions.

## Introduction

Several studies provide evidences indicating that hippocampal neurogenesis is needed for the integration of new information into pre-existing context promoting flexible learning and adaptive behaviors. Physiological experiences such as learning, physical exercise and exposition to enrich environment (EEs) have been associated with an increase in survival, proliferation and differentiation of adult-born hippocampal cells. Moreover, others pathophysiological conditions, such as aging, stress, and degenerative disorders (like Alzheimer disease, AD) have been described to impair and decrease adult neurogenesis (Goncalves et al., 2016; Toda et al., 2019). These effects are modulated through different signaling molecules produced in the adult hippocampal neurogenic niche.

Understanding the signals derived from this specific microenvironment results essential to enhance the process of neuronal integration in the aged and diseased brain. In this minireview, we focus our attention in the complexity of the adult hippocampal neurogenic niche, which provides multiple signals that are integrated by the neural stem cells (NSCs) and the newborn neurons to respond adequately in different circumstances.

## Adult Hippocampal Neurogenesis

Adult hippocampal neurogenesis has been confirmed in the majority of mammals, but whether it is present in humans has been the issue of an intense recent debate (Boldrini et al., 2018; Sorrells et al., 2018). Methodological factors seem to contribute to the discrepancies between studies that describe the presence or absence of neurogenesis in the human adult dentate gyrus (DG). Future research using different approaches will be needed to understand how adult-born granule cells (GCs) are generated. Recent studies describe that human hippocampal neurogenesis persists through the ninth decade of life and is associated with cognitive status in patients with AD, providing evidence of the potential relevance of this process for many human disorders (Moreno-Jimenez et al., 2019; Tobin et al., 2019).

The general pattern of hippocampal neurogenesis is conserved across different mammalian species. Hippocampal NSCs give rise to GCs throughout a highly regulated process, which involves the exit of the quiescence state, posterior divisions, specification to a neuronal fate, neuronal differentiation, and the physiological integration in the preexisting hippocampal circuits. Along this period morphological, intrinsic electrical properties and synaptic connections evolve in parallel toward a mature neuronal phenotype. All the process is tightly controlled by physiological stimuli, that modify the hippocampal niche (Toni and Schinder, 2015; Toda et al., 2019).

## Cellular Components of the Hippocampal Neurogenic Niche

**FIGURE 1:**
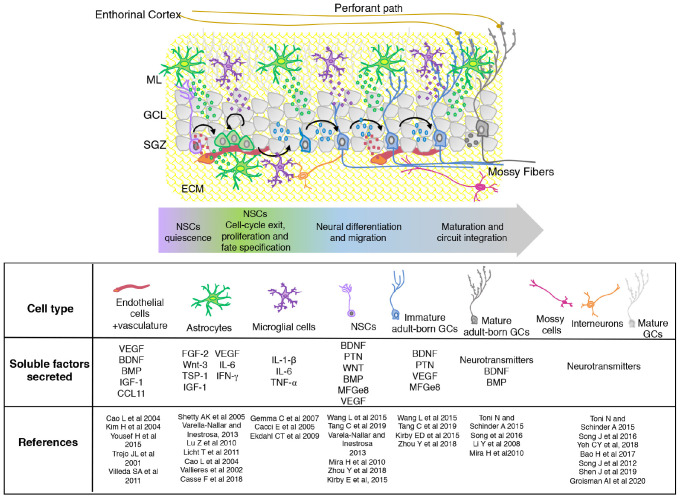
Scheme showing the organization and composition of the adult hippocampal neurogenic niche. The different stages of adult born GCs maturation are shown with neuronal and non-neuronal (astrocytes, microglia, and vascular cells) components. The extracellular matrix (ECM) is indicated in yellow. Soluble diffusible signaling molecules produced by the different cellular components of the SGZ niche are mentioned in the table. SGZ, subgranular zone; GCL, granular cell layer; ML, molecular layer.

The adult hippocampal neurogenic niche is a specialized and dynamic microenvironment, which involves both cellular and non-cellular components of the DG. Altogether, cells and the signals produced by them can regulate the neurogenic process acting at different levels from proliferation to functional integration (Figure 1).

### Astrocytes

Astrocytes represent one of the main modulators of the neurogenic niche (Song et al., 2002). They control cell proliferation, migration, differentiation and synaptic integration of newborn GCs through membrane-associated molecules and by secreting soluble signals like fibroblast growth factor-2 (FGF-2), WNT (Wingless) ligands, thrombospondin-1 (TSP-1), cytokines, and extracellular matrix (ECM) proteins among others (Trejo et al., 2001; Shetty et al., 2005; Lu and Kipnis, 2010; Casse et al., 2018). They also control the availability of neurotransmitters in the synaptic cleft. The relevance of astrocytes in the maturation of adult-born GCs was evidenced using transgenic approaches to block vesicular release. This strategy resulted in both reduced glutamatergic synaptic input and dendritic spine density that was accompanied by a reduction in cell survival and functional integration of adult-born, but not of mature DG neurons (Sultan et al., 2015). Astrocytes can affect positively or negatively neurogenesis, depending on their metabolic state. While in normal physiological conditions astrocytes produce molecules that positively regulate this process, in pathological situations, they suffer modifications in their transcriptome and secretome that may contribute to impairment of neurogenesis and cognitive deficits. Thus, cytokines such as IL-6, TNF-α, and IFN-γ are produced by astrocytes in inflammatory processes (Vallieres et al., 2002; Liddelow and Barres, 2017; Casse et al., 2018).

### Microglia

Several studies have shown the relevance of microglia in adult hippocampal neurogenesis. They are involved in phagocytosis of apoptotic adult-born GCs (Sierra et al., 2010). Therefore, ablation of microglia in the adult DG results in decreased number of neuroblasts (Kreisel et al., 2019). Interestingly, a recent report has described that phagocytic microglia act as a sensor of local cell death and modulate the balance between cell proliferation and cell survival in the neurogenic niche (Diaz-Aparicio et al., 2020). Microglial cells regulate neurogenesis through both cell-cell interaction mechanisms and secreted factors. Thus, animals lacking CX3CR1 microglial receptor, involved in microglial-neuronal interaction, resulted in impaired morphology and deficient synaptic integration of adult-born GCs in the DG (Bolos et al., 2018). Microglial activation by pro-inflammatory molecules results in defects in different steps of adult neurogenesis. Cytokines secreted by microglia in the context of inflammation include: IL-6, IL-1β, and tumor necrosis factor-α (TNF-α; Cacci et al., 2005; Gemma et al., 2007; Ekdahl et al., 2009).

### Vascular Cells

A growing body of data indicates that blood vessels are essential components of hippocampal NSC niches. Vascular cells can impact neurogenesis directly by producing neurogenic factors or indirectly, transporting neurogenic substances produced by other cells. Many studies indicate that endothelial cells secrete different trophic factors such as brain-derived neurotrophic factor (BDNF), vascular endothelial growth factor (VEGF), and chemokines such as CCL11, which affect NSCs proliferation and maturation of these cells (Cao et al., 2004; Kim et al., 2004; Licht et al., 2011; Villeda et al., 2011; Licht and Keshet, 2015). A recent study indicates that endothelial cells, through the expression of the monocarboxylic acid transporter 1 (MCT1), contribute to the maintenance of lactate homeostasis promoting neurogenesis and cognitive functions (Wang et al., 2019). Another important source of neurogenic signals comes from the brain vasculature which provide signaling molecules secreted by local or distal sources. These include trophic factors, hormones, lipids and exosomes (Batiz et al., 2015; Licht and Keshet, 2015).

### Neural Stem Cells and Neuronal Cells

Increasing evidence shows an important role for NSCs as regulators of their own niche, influencing the development of their progeny at different neurogenic stages. VEGF, neurotrophin-3 (NT3), Pleiotrophin (PTN), and BDNF are some of the factors released by the NSCs (Vicidomini et al., 2020).

Neuronal activity regulates multiple stages of adult neurogenesis from proliferation, survival, neuronal maturation, and synaptic integration. Local interneurons, hilar inhibitory neurons, mossy glutamatergic neurons and mature GCs from the DG control different stages of newborn GCs integration. Extensive literature has demonstrated an essential role of neurotransmitters locally released by DG neurons or by axons arising from projecting neurons in the modulation of adult-born GCs development (Song et al., 2012, 2016; Toni and Schinder, 2015; Bao et al., 2017; Yeh et al., 2018; Groisman et al., 2020). This topic will not be discussed in the present revision.

The cellular components of the subgranular zone (SGZ) provide a complex regulatory architecture that allow the correct development of the adult-born GCs, promoting their correct integration in the preexisting hippocampal circuits. Neural activity triggered by physiological experiences is essential to govern the interaction between the different cellular components that control the neurogenic process by secreting specific signals. An interesting example of the signal integration in the hippocampal neurogenic niche was evidenced in a recent study which shows that hippocampus-associated behaviors increase microvascular blood-flow velocity in the DG and enhance hippocampal neurogenesis. The authors proved that this effect is mediated by parvalbumin-expressing neurons which increase blood flow via nitric-oxide signaling. This increase in the microvascular hemodynamics enhances IGF-1 signaling promoting the newborn cell survival (Shen et al., 2019).

## Subgranular Zone Niche Signals

**FIGURE 2:**
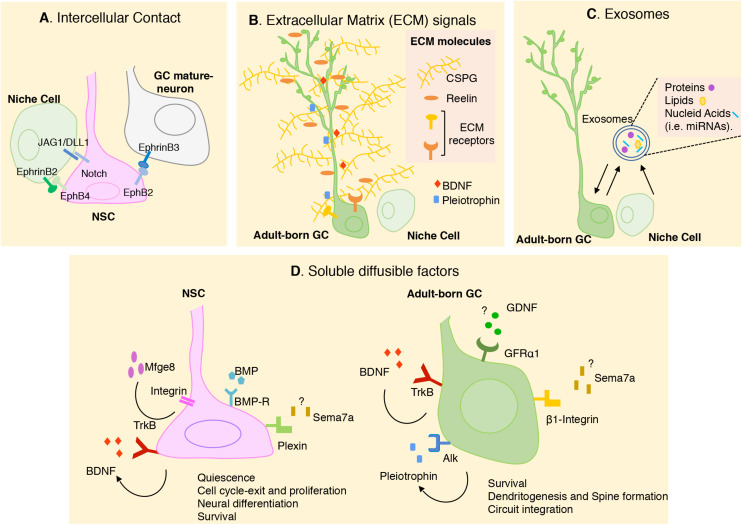
Schematic representation of the different cellular and molecular mechanisms that modulate adult neurogenesis in SGZ. In the figure, we summarize the novel signals most recently described. **(A)** Intercellular contacts including Notch/JAG1/DLL1 and Eph/ephrines between NSCs and adjacent cells. **(B)** Extracellular matrix (ECM) molecules contributes to the preservation of stem cells pool and the morphological differentiation of adult born-GCs. ECM can also modulate the availability of soluble factors present in the SGZ niche, like Pleiotrophin (PTN) and BDNF. **(C)** Exosomes has recently been proposed to have a key role in cell-cell communication in SGZ niche. **(D)** Soluble diffusible factors have been described to have multiple roles in regulating adult hippocampal neurogenesis. Some external signals, their receptors and their biological action were indicating. Question mark indicates that the source of the ligand is still unknown. Arrow indicates autocrine signaling. More studies are needed to understand the interaction between these signals. GC, granular cell; NSC, neural stem cell; CSPG, chondroitin sulfate proteoglycan.

The different cellular components of the neurogenic niche can modulate neurogenesis by multiple signaling mechanisms (Figure 2). Here we describe different types of signals produced by the SGZ niche focusing in the new advances and novel factors that has been described during the last years.

### Intercellular Contacts

Direct cell–cell interaction is critical in stem cell maintenance. A known membrane molecule, Notch, and its ligands can mediate direct interaction between NSCs and neighboring cells, and thus play an important role in neurogenesis. Ablation of Notch in hippocampal NSCs during adulthood promotes cell cycle exit and neuronal fate determination (Breunig et al., 2007; Ables et al., 2011). The importance of Notch signaling in the maintenance of NSC quiescence in SGZ has been also demonstrated by ablation of the Notch ligands DELTA1 (DLL1) and JAGGED1 (JAG1) in DG stem cells (Ehm et al., 2010; Imayoshi et al., 2010; Kawaguchi et al., 2013; Lavado and Oliver, 2014). Notch ligands are also expressed by astrocytes from the adult DG and reduction in the levels of JAG1 results in a reduction in Notch signaling and increase in neuronal differentiation (Wilhelmsson et al., 2012).

Eprhrin/Eph signaling has also been involved as important players regulating stem cell behavior. Initial studies showed that Ephrin-B2 presented by astrocytes interacts with EphB4 receptors on NSCs, promoting neuronal differentiation (Ashton et al., 2012). A recent study indicates that the intercellular signaling between mature GCs and NSCs regulates the transition of quiescent NSCs to newborn neurons. During running, membrane-bound ligand, Ephrin-B3 on mature GCs acts as a negative regulator for activation of adjacent NSCs expressing EphB2 receptor (Dong et al., 2019).

### Extracellular Matrix Signals

All cell types in the SGZ niche are in contact with the ECM, a complex and dynamic network of macromolecules with different physical and biochemical properties. The ECM acts providing a physical supportive structure and also molecular signals to regulate NSC development. The contribution of the ECM molecules to the modulation of hippocampal neurogenesis is complex, as they can act by interacting directly with cellular receptors or indirectly as modulators of the availability of soluble factors present in the neurogenic niche (Figure 2B). Among ECM molecules that have been involved in hippocampal neurogenesis is the extracellular glycoprotein Reelin, which promotes NSC proliferation and also dendritic maturation (Won et al., 2006; Teixeira et al., 2012). During the last years proteoglycans have emerged as important cues for the proliferation and differentiation of new neurons in the SGZ. Thus, pharmacological depletion of chondroitin sulfate proteoglycan (CSPG) in the DG reduces the densities of newborn GCs. The dendritic arborization of these neurons was also reduced by CSPG digestion, and behavioral analysis of these animals revealed cognitive memory impairments. Interestingly, the ability of EE to promote GC production and improve cognitive behaviors was impaired in mice that lacked a key enzyme for CSPG synthesis indicating that the extracellular CSPGs participate in the pro-neurogenic effects of the EE (Yamada et al., 2018). Another major constituent of the forebrain ECM is the glycosaminoglycan hyaluronan (Hyaluronic acid, HA), which is present in the SGZ. Mice lacking the HA transmembrane receptor, CD44, which is expressed by NSC, show an increase in stem cell proliferation, suggesting a role of this molecule in NSC quiescence. The fact that HA is synthesized by NSC and increases in the SGZ with aging suggest that HA accumulation may contribute to the reduced neurogenesis observed in aged animals (Su et al., 2017).

### Soluble Diffusible Factors

The different cells that constitute the DG neurogenic niche regulate stem cell activity by secreting diffusible signaling molecules, which represent the majority of extracellular cues that regulate neurogenesis (Figure 2D). Among them, the role of bone morphogenetic proteins (BMPs) and WNT signaling has been well established. Thus, WNT signaling produced by NSCs and astrocytes in the SGZ can regulate different stages of adult neurogenesis. It is well-known that WNT signaling promotes proliferation and NSC self-renewal, while, endogenous WNT signaling inhibitors, such as sFRP3 and Dkk1, promote stem cell quiescence and controls the timing of newborn granule neuron maturation (Lie et al., 2005; Bowman et al., 2013; Jang et al., 2013; Seib et al., 2013; Varela-Nallar and Inestrosa, 2013). Different members of the WNT family have also been associated to the promotion of dendrite development of adult born GCs (Arredondo et al., 2020). Regarding to the BMPs, they have emerged as critical inducers of NSC quiescence and long-term maintenance in SGZ (Gobeske et al., 2009; Mira et al., 2010; Yousef et al., 2015). The soluble factor Sonic Hedgehog (Shh), which is critical at early stages of embryonic brain development, has also been involved in adult hippocampal neurogenesis promoting the proliferation SGZ NSCs before they become quiescent (Han et al., 2008; Noguchi et al., 2019).

Trophic factors, such as IGF-1 and VEGF are relevant players involved in adult neurogenesis at different developmental stages that have previously been deeply analyzed (Cheng et al., 2001; Lichtenwalner et al., 2001; Fournier and Duman, 2012; Kirby et al., 2015; Nieto-Estevez et al., 2016; Mir et al., 2017).

During the last years new soluble molecules known for other functions, have emerged as modulators of the neurogenic process. Thus, the Globule-epidermal growth factor (EGF) 8 (MFGe8), a molecule involved in the phagocytosis of apoptotic cells, was found to be expressed by quiescent NSCs and astrocytes in the SGZ. Recently, it was shown that adult specific deletion of MFGe8 in NSCs promotes the increase in NSC proliferation and depletion of the neurogenic pool causing a decreased neurogenesis at later developmental stages (Zhou et al., 2018). Another soluble protein, Semaphorin7a (Sema7a), which has been previously described as a guidance molecule, has emerged as a novel key factor in the control of adult hippocampal neurogenesis. Interestingly, Sema7a regulates different stages of adult neurogenesis via two, stage-specific different receptors. Thus, Sema7a inhibits progenitor proliferation by acting though Plexin, in early neural progenitors and subsequently, during differentiation, Sema7a promotes dendrite maturation and spine development acting through β1-integrin receptors (Jongbloets et al., 2017).

The role of the neurotrophins in hippocampal adult neurogenesis is well documented. Particularly, BDNF is expressed in SGZ by NSCs, mature DG granule neurons and also by non-neuronal cells, while its receptor, TrkB, is broadly expressed by NSCs at different developmental stages (Vilar and Mira, 2016). Brain-derived neurotrophic factor acting through TrkB has been associated to survival, proliferation and maturation of adult-born GCs (Scharfman et al., 2005; Li et al., 2008; Taliaz et al., 2010). Dendrite development, spine growth and synapse formation were markedly impaired in adult-born GCs from TrkB-deficient mice in which the receptor was conditionally deleted in NSC and in animals in which BDNF was ablated in the entire forebrain (Bergami et al., 2008). Interestingly, conditional deletion of BDNF in NSCs resulted in a similar impairment in dendrite growth indicating that the effect of BDNF on dendrite maturation is mainly autocrine. In support of an autocrine role of BDNF, its deletion in NSC abolished the promotion of dendritic growth induced by running (Wang et al., 2015).

Other member of the neurotrophin family, NT-3 is highly expressed in the adult DG. Conditional ablation of NT-3 in the brain throughout development shows normal proliferation in the SGZ, a reduction in the number of newly generated granule neurons and an increase in the proportion of cells that do not express differentiation markers, indicating a role of NT3 in maturation of neural progenitor cells (Shimazu et al., 2006).

A more recent work has demonstrated that the protein PTN secreted by hippocampal NSCs from the SGZ niche is important for the correct development and integration of the new neurons in the DG. Ablation of PTN leads to defects in neuronal integration and synaptic activity of the newborn neurons in the hippocampus without affecting the production or survival of them. This effect is mediated by one of the PTN receptors, ALK, which is expressed by NSCs. Interestingly, this study showed that the expression of PTN is reduced with aging but that the administration of PTN is able to ameliorate the age-induced defects of hippocampal neurogenesis (Tang et al., 2019).

Recently, glial-derived neurotrophic factor (GDNF), a neurotrophic factor initially described for its potent effect on the survival of dopaminergic nigrostriatal neurons was described as a novel regulator of newborn GCs integration (Paratcha and Ledda, 2008; Bonafina et al., 2019). The receptor of GDNF, the GPI-linked protein GFRα1, is expressed by immature and mature adult-born GCs. Conditional ablation of GFRα1 in NSCs indicated that GDNF/GFRα1 complex is required for proper maturation and integration of adult-born GCs into preexisting hippocampal circuits. Conditional knockout mice for GFRα1 showed impairment in behavioral pattern separation, which has been associated to deficits in adult neurogenesis. This study shows that voluntary physical exercise promotes GDNF expression in the DG and dendritic development. However, the deletion of GFRα1 in the newborn GCs abolishes the increase in dendrite complexity induced by running, revealing that the effect of running on dendrite development depends partially on GDNF expression (Bonafina et al., 2019).

As growth factors involved in hippocampal neurogenesis acts through different receptors triggering specific downstream signaling pathways, the remaining question is how newborn neurons integrate this information. One possibility is that the same cell expresses all the receptors but need to integrate the different signals in order to respond appropriately. A second possibility is the existence of subpopulations of adult-born GCs each of which respond to different growth factors expressing specific receptor repertoires. Moreover, the presence and the abundance of receptors and the downstream signaling partners can be modified during the maturation process. Thus, the expression of different arrays of trophic factor receptors in the adult-born GCs deserve further analysis.

### Exosomes

These small membrane extracellular vesicles have emerged as one of the major mediators of intercellular communication (Figure 2C). Diverse array of proteins, lipids, mRNAs and miRNAs have been identified in exosomes from different cell types found in the SGZ niche. Although the role of exosomes in the adult neurogenic niches is still unclear, growing indirect evidence suggest that exosomes might play a critical role in cell-cell communication in neurogenic niches (Batiz et al., 2015). Some of the molecules expressed in the neurogenic environments have been reported to be present in exosomes. Recently, a study has shown that injection of purified exosomes derived from neural cultures in postnatal mouse brains increases SGZ neurogenesis indicating that exosomes contain molecular cargo that regulates this process (Sharma et al., 2019).

### Lipids

Over the last years, lipids have gained attention in the regulation of adult neurogenesis (Knobloch, 2017). Lipids can be taken up from circulation or synthetized de novo by NSCs. Cholesterol-carrying lipoproteins receptor, LDL-r, has been associated to adult hippocampal neurogenesis. Ablation of LDL-r in mice results in a reduction of the proliferation of NSCs and also a decline in the number of newborn GCs. These results were confirmed by in vitro experiments in which NPCs exposed to high concentration of plasma LDL results in a decreased proliferation and reduced differentiation toward a neuronal lineage (Engel et al., 2019). Although several studies indicate the relevance of lipids in neurogenesis, how lipids affect this process needs to be addressed in more detail.

The large literature about the different cells and the nature of signals which modulate adult hippocampal neurogenesis indicates that extrinsic control of this process is much more complex than previously envisioned. The distribution of different factors in the neurogenic niche, the precise signaling pathways that they trigger, the interaction with other intrinsic and extrinsic signals and their function in pathological process deserves further investigation.

## Discussion

The great diversity of signals present in the niche should be appropriately integrated by the adult-born GCs to promote the proper maturation and integration of them into preexisting circuits. The different factors derived from the microenvironment induce specific transcriptional programs that drive the maturation of the new cells and determine the morphological and physiological properties of GCs at the different stages during neuronal development and their response to external stimuli.

In parallel to the great diversity of signals that have been described as modulators of the hippocampal neurogenesis, different studies pointed out to the heterogeneity of NSCs. This idea indicates that not all NSCs or immature GCs respond similarly to the different extracellular signals that are present in the niche (Shin et al., 2015). Cellular heterogeneity in these neurons may result in some populations being more responsive to the variety of factors present in the niche and also being more susceptible to different pathologies.

The identification of the array of factors present in the SGZ niche during neurogenesis represents a crucial knowledge because it opens the possibility to combine them in order to improve the development of adult-born neurons in physiopathological conditions. In this context a recent study reported that mimicking the beneficial effects of exercise by pharmacological induction of neurogenesis, combined with elevation of BDNF levels in the DG revert the negative effects of Alzheimer’s disease on newborn hippocampal neurons in a mouse model of the disease (Choi et al., 2018).

Thus, understanding the complexity of the SGZ neurogenic niche becomes essential for the development of novel therapeutic strategies for the treatment of cognitive impairments associated with aging and brain disorders in which adult hippocampal neurogenesis is affected.

## Author Contributions

All authors contributed equally to this mini-review.

## Conflict of Interest

The authors declare that the research was conducted in the absence of any commercial or financial relationships that could be construed as a potential conflict of interest.
